# Remarkable Response of *EGFR-* and *HER2-*Amplified Metastatic Colon Cancer to Pyrotinib After Failed Multiline Treatments: A Case Report and Literature Review

**DOI:** 10.3389/fonc.2020.548867

**Published:** 2020-10-26

**Authors:** Hong-Shuai Li, Li-Li Yang, Ming-Yi Zhang, Ke Cheng, Ye Chen, Ji-Yan Liu

**Affiliations:** ^1^Department of Biotherapy, Cancer Center, and National Clinical Research Center for Geriatrics, West China Hospital, Sichuan University, Chengdu, China; ^2^Sichuan Clinical Research Center of Biotherapy, Chengdu, China; ^3^Department of Medical Oncology, Chengdu Shangjinnanfu Hospital/West China Hospital of Sichuan University, Chengdu, China; ^4^Department of Abdominal Oncology, West China Hospital of Sichuan University, Chengdu, China

**Keywords:** colorectal cancer, human epidermal growth factor receptor 2, trastuzumab, resistance, pyrotinib

## Abstract

Human epidermal growth factor receptor 2 (HER2) has been verified as a valuable biomarker and treatment target in metastatic colorectal cancer (mCRC). Pyrotinib, a novel irreversible HER2/epidermal growth factor receptor (EGFR) dual tyrosine kinase inhibitor, can efficiently inhibit the proliferation of HER2-positive cancer cells in many tumors. We report a case of a 40-year-old woman with both HER2- and EGFR-amplified metastatic colon cancer, who developed refractory disease resistant to multiline therapies (including trastuzumab with lapatinib) but achieved a remarkable response after pyrotinib treatment. For patients with HER2-positive mCRC, who have developed resistance to trastuzumab and lapatinib, pyrotinib is a promising new candidate, which can be used as salvage therapy.

## Introduction

Application of targeted therapy against epidermal growth factor receptor (EGFR) (e.g., cetuximab and panitumumab) and vascular endothelial growth factor (VEGF) (e.g., bevacizumab) has brought survival benefit for metastatic colorectal cancer (mCRC) patients over the past few years. However, many patients will experience unavoidable progressive disease (PD) after receiving 2 or more lines of treatment, and those patients achieving objective responses of currently recommended third-line therapies such as the multi-kinase inhibitor regorafenib, tipiracil combination TAS-102 are limited. Later-line treatments for mCRC still remain a challenge.

Overexpression of human epidermal growth factor receptor 2 (HER2) in mCRC is only detected in 2–6% of cases (1), but is more common in RAS/BRAF wild-type tumors (5–14%) (2, 3). According to the most recent National Comprehensive Cancer Network (NCCN) guidelines, trastuzumab plus [lapatinib or pertuzumab] is already recommended as subsequent-line therapy for HER2-amplified and RAS/BRAF wild-type mCRC after the failure of chemotherapy. However, no standard of care for trastuzumab-resistant mCRC was recommended. Pyrotinib is a novel irreversible EGFR/HER2 dual TKI (4) that efficiently inhibits the proliferation of HER2-positive cancer cells in many tumors, including breast cancer (5) and lung cancer (6). Pyrotinib has been shown to be effective in a phase II randomized controlled trial (7) and was then approved for the treatment of HER2-positive recurrent or metastatic breast cancer by the Chinese Food and Drug Administration. Currently, it is unclear whether pyrotinib is effective in trastuzumab-resistant mCRC.

Herein, we report a HER2-amplified mCRC case, who had previously been treated with trastuzumab and lapatinib, benefiting from pyrotinib.

## Case Presentation

**Figure 1:**
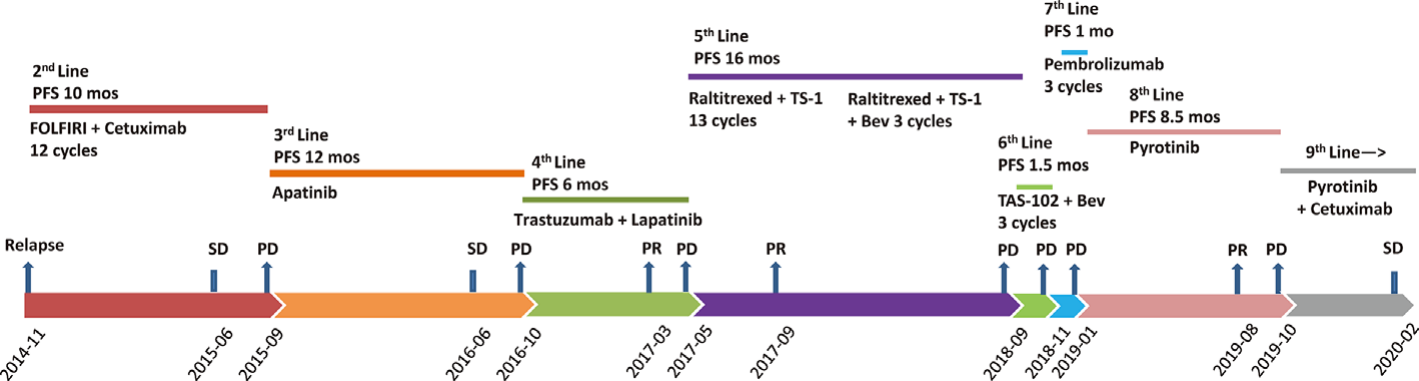
Treatment process after post-operative relapse. PFS, progression-free survival; FOLFIRI, irinotecan, leucovorin, and fluorouracil; Bev, bevacizumab; PD, progressive disease; SD, stable disease; PR, partial response; mos, months.

**Figure 2:**
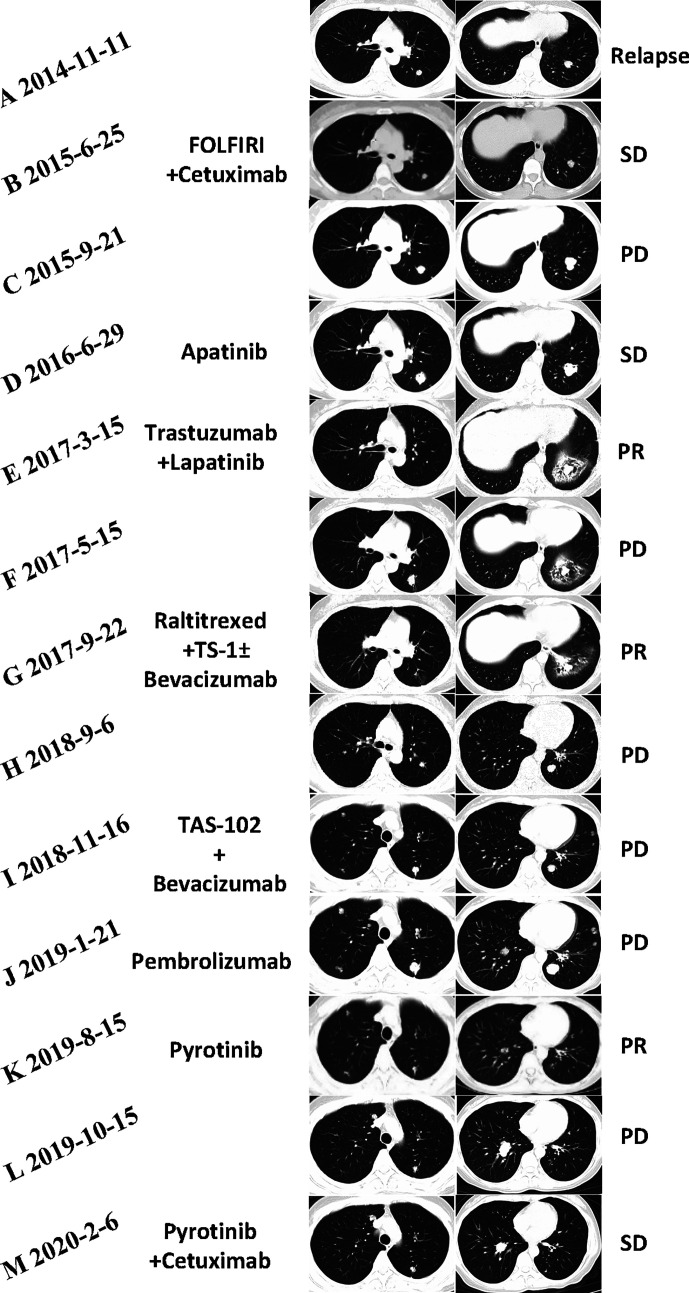
**(A)** Initial lung metastases were revealed by CT (2014-11). **(B)** The best efficacy achieved by the combination of FOLFIRI (irinotecan, leucovorin, and fluorouracil) and cetuximab was stable disease (SD) (2015-6). **(C)** CT (2015-9) demonstrated progressive disease (PD) in the lung metastases when first-line chemotherapy failed. **(D)** The SD status (2016-6) achieved by apatinib had not been changed until a cerebellum metastasis (images unavailable) was found in October 2016. **(E)** CT (2017-3) showed that the lung lesions had markedly shrunken in size 4 months after trastuzumab and lapatinib. **(F)** After 6 months of trastuzumab and lapatinib treatment as third-line therapy, CT (2017-5) suggested new lung metastases. **(G)** CT scans revealed PR after treatment of raltitrexed plus TS-1 plus bevacizumab for 4 months. **(H)** CT scans (2018-9) showed PD in pulmonary metastatic lesions again. **(I)** Regimen of TAS-102 and bevacizumab failed after 3 cycles. **(J)** CT scans (2019-1) revealed that lung lesions are increased and enlarged significantly after treatment with pembrolizumab for 3 cycles. **(K)** The number and size of pulmonary metastases considerably decreased after the treatment of single pyrotinib for 6 months. **(L)** CT scan (2019-10) showed PD in the lung lesions again. **(M)** CT scan demonstrated SD of the lung lesions after the treatment of pyrotinib plus cetuximab for 4 months.

In April 2013, a previously healthy 40-year-old woman complaining of persistent pain in the left upper abdomen was admitted to our hospital. The patient did not have a significant family history of cancer. No significant abnormalities was found in laboratory tests including tumor markers (carcinoembryonic antigen, 2.8 ng/ml; CA, 19-9 7.5 IU/ml). T2-weighted magnetic resonance imaging (MRI) of the upper abdomen revealed colon and liver lesions. A radical colectomy combined with liver lesions resection was conducted on May 13, 2013. Pathological examination confirmed the presence of moderately differentiated adenocarcinoma of splenic flexure (pT3N1cM1a IVA), with no mutations in *RAS* and *BRAF* but strong expression of HER2 (IHC). Adjuvant chemotherapy with the mFOLFOX6 regimen (oxaliplatin, leucovorin, and fluorouracil) was given for 12 cycles following the surgery. **Figure 1** shows the entire treatment process, and **Figure 2** shows the corresponding changes in the lung lesions during treatment on computed tomography (CT) scan. Laboratory tests results including tumor markers were not listed due to no obvious abnormalities throughout the treatment process.

### Second-Line Chemotherapy Combined With Cetuximab

On November 11, 2014, a chest CT scan revealed lung metastases; given that the end of adjuvant chemotherapy of mFOLFOX6 was only 10 months from relapse (<12 months), an alternative regimen of FOLFIRI (irinotecan, leucovorin, and fluorouracil) plus cetuximab was given instead. However, after 12 cycles of chemotherapy with a period of stable disease (SD), the efficacy of PD was shown by the chest CT scan on September 21, 2015.

### Apatinib Monotherapy

As both regorafenib and TAS-102 were not available at that time in China, the patient was then treated with apatinib (750 mg, orally, once daily) as third-line therapy from October 2015. During the treatment with apatinib, the patient developed grade 2 adverse effects of hand-foot skin reaction after the first month of medication and aggravated to grade 3 after two months. The dose of apatinib was then reduced to 500 mg, once daily. A three-month routine chest CT scan indicated SD of lung lesions until October 2016, when the patient suddenly felt dizzy and vomited, and a head enhanced-MRI showed a 5×4 cm mass located in the cerebellum area. The patient then underwent a resection of the cerebellum mass which was later confirmed as metastasis of colon cancer by pathological examination. Subsequently, next-generation sequencing (NGS) (451 genes; HaploX Biotechnology Co., Ltd, Shenzhen, China) was performed on the cerebellum specimen. HER2 amplification was confirmed by the NGS. The detailed results are listed in **Table 1**.

**Table 1:** Gene alterations before and after treatment of trastuzumab and lapatinib by NGS.

Genes	Before trastuzumab and lapatinib			After trastuzumab and lapatinib		
	Variations	Copy number	Abundance	Variations	Copy number	Abundance
*KRAS*	Negative	–	–	Amplification	2.2	–
*HRAS*	Negative	–	–	Negative	–	–
*NRAS*	Negative	–	–	Negative	–	–
*BRAF*	Negative	–	–	Negative	–	–
*EGFR*	Amplification	3.4	–	Amplification	2.4	–
*HER2*	Amplification	27.7	–	Amplification	14.3	–
*HER4*	V781A	–	20.61%	Negative	–	–
*NTRK1*	Negative	–	–	Negative	–	–
MSI	MSI-H	–	–	MSI-H	–	–
TMB	Moderate	–	10.4/Mb	High	–	95/Mb
*TP53*	R175H	–	21.86%	R175H	–	9.37%
	R306*	–	65.48%	R267X	–	5.30%
*BRCA2*	Negative	–	–	R2108H	–	12.81%
*PTEN*	Negative	–	–	Negative	–	–
*PIK3CA*	Negative	–	–	Negative	–	–
*INPP4A*	Negative	–	–	T666I	–	25.14%
*INPP4B*	Negative	–	–	Del LOH	1.5	–
*FGFR1*	Amplification	4.6	–	Amplification	2.7	–
*FGFR2*	Negative	–	–	M186T	–	30.9%
*BRCA2*	Negative	–	–	R2108H	–	12.81%

KRAS, kirsten rat sarcoma viral oncogene homolog; HRAS, harvey rat sarcoma viral oncogene homolog; NRAS, neuroblastoma rat sarcoma viral oncogene homolog; BRAF, v-raf murine sarcoma viral oncogene homolog B1; EGFR, epidermal growth factor receptor; HER2, human epidermal growth factor receptor 2; HER4, human epidermal growth factor receptor 4; NTRK1, neurotrophic tyrosine kinase, receptor, type 1; MSI, microsatellite instability; MSI-H, microsatellite instability-high; TMB, tumor mutational burden; TP53, tumor protein p53; BRCA2, breast cancer susceptibility gene 2; PTEN, phosphatase and tensin homolog; PIK3CA, phosphoinositide-3-kinase, catalytic, alpha polypeptide; INPP4A, inositol polyphosphate-4-phosphatase, type I, 107 kDa; INPP4B, inositol polyphosphate-4-phosphatase, type II, 105 kDa; FGFR1, fibroblast growth factor receptor 1; FGFR2, fibroblast growth factor receptor 2; BRCA, breast cancer gene; NGS, next generation sequencing.

### Trastuzumab Combined With Lapatinib

Based on the NGS results, a regimen of trastuzumab combined with continuous lapatinib (1.0 g, orally, once daily) was then initiated from October 2016. The patient was well tolerated with this new protocol without obvious adverse effects. Four months later, the CT scan showed that no relapse was detected in the cerebellum area and the lung lesions had markedly shrunken in size with a therapeutic evaluation as partial response (PR).

### Raltitrexed Plus TS-1 Combined With Bevacizumab

In May 2017, a follow-up CT scan revealed a new 1.8×2 cm metastatic lesion in the lower lobe of left lung. Raltitrexed plus TS-1 was applied as a salvage regimen subsequently. Five cycles later, this new lesion disappeared on the chest CT scan carried out in September 2017 and the patient obtained an efficacy of PR again. This PR status was sustained until July 2018 when the chest CT indicated that the lung lesions are slightly larger than before but not to the extent of PD. Thus, we added bevacizumab on the basis of the original plan. Three cycles later, the CT suggested PD by showing more and larger lung lesions than before. In September 2018, the patient was administered with TAS-102 and bevacizumab; however, this combination failed after three cycles.

### Pembrolizumab Monotherapy

A biopsy of the lung metastasis was conducted and NGS (340 genes; Euler Genomics Co. Ltd., Beijing, China) was performed subsequently. The results yielded the following mutation profile: *HER2*^amp^, *EGFR*^amp^, *INPP4A*^T666I^, *INPP4B*^del^ ^LOH^, *KRAS*^amp^, *BRCA2*^R2108H^, *FGFR1*^amp^, MSI-H, and a high TMB of 95 muts/Mb (**Table 1**). Immunohistochemistry was negative for PD-1/PD-L1 expression. With available therapies exhausted, the patient applied pembrolizumab as salvage treatment from December 2018. However, only one month later, the CT scan revealed that the tumors in the lungs had increased and enlarged significantly.

### Pyrotinib Monotherapy

In January 2019, after receiving written informed consent, we changed the regimen to pyrotinib at a dose of 400 mg, orally, once daily. Confirmed partial response (PR) was observed during CT re-examination in August 2019, with impressive reductions in both the numbers and size of the lung lesions (**Figure 2K**). The patient tolerated treatment with pyrotinib well. No additional adverse events except grades 1 to 2 vomiting and diarrhea were observed.

### Rechallenge of Cetuximab Combined With Pyrotinib

In October 2019, the CT scan showed PD in the lung lesions again. Re-biopsy of the progressive lesions was recommended, but the patient refused. She was finally rechallenged with cetuximab combined with pyrotinib (pyrotinib 400 mg, once daily; cetuximab 800 mg, every 2 weeks). The patient developed grade 3 acneiform rash during the medication, and minocycline (100 mg, once daily) was thus prescribed for the control and symptomatic relief. After 2 weeks, this symptom gradually subsided to grade 2 and was well tolerated by the patient. On February 6, 2020, the CT scan demonstrated SD of the lung lesions (**Figure 2M**), and the patient had a Karnofsky score of 80 and a QOL (quality of life) score of 51. Until the submission of the case draft, the patient has survived for over 63 months since post-operative recurrence and now continues to receive the combination treatment of pyrotinib and cetuximab and the PFS is over 4 months.

## Discussion

To date, mutations in KRAS and NRAS and the BRAF V600E mutation, which are of great value in determining clinical prognosis and guiding patient treatment strategies, have been studied extensively in mCRC, resulting in the establishment of standard-of-care testing. Moreover, mismatch repair deficiency (dMMR)/microsatellite instability (MSI) and TMB status are also important indicators of patient prognosis and can facilitate treatment choice, especially for the application of immune checkpoint blockade (1). CRC is driven by various oncogenes, and novel treatments targeting tumor driver genes in CRC, including targeting of HER2 (**Supplementary Table 1**), are being studied nowadays. A literature review was conducted to identify the latest clinical trials (with results released) exploring the medical efficacy of anti-HER2 therapy in patients with mCRC (**Table 2**). An emerging scenario of anti-HER2 therapy was revealed: trastuzumab plus [lapatinib or pertuzumab], pertuzumab plus T-DM1, and trastuzumab plus tucatinib. Eligible studies were identified through multiple databases (EMBASE, Web of Science, PubMed, the American society of clinical oncology (https://www.asco.org/), the European society for medical oncology (https://www.esmo.org/), the National Institutes of Health trial registry (Clinicaltrials.gov), and the EU Clinical Trials Register (clinicaltrialsregister.eu). Relevant studies were searched for using relevant medical terms for subject headings (“metastatic colorectal cancer,” “advanced colorectal cancer,” “HER2,” “phase,” and “clinical trial”) and abstracts (“metast,” “HER2,” “neoplasm metastasis,” “neoplasm recurrence,” and”colorectal cancer”).

**Table 2:** Clinical trials targeting HER2 for metastatic colorectal cancer.

Study	Intervention	Number	ORR	DCR	PFS, mo	OS, mo	Phase
HERACLES	Trastuzumab plus lapatinib	27	30%	59%	4.9	10.7	II
Mypathway	Trastuzumab plus pertuzumab	57	32%	60%	2.9 (KRAS WT 5.3; KRAS MT 1.4)	11.5 (KRAS WT 14.0; KRAS MT 8.5)	IIa
HERACLES-B (LBA 35)	Pertuzumab plus T-DM1	30	10%	80%	4.9	–	II
TRIUMPH (526 PD)	Trastuzumab plus pertuzumab	18	35%	65%	4.0	–	II
MOUNTAINEER (527 PD)	Trastuzumab plus tucatinib	23	52%	91%	8.1	18.7	II
DESTINY-CRC01	DS-8201	90	45%	83%	6.9	–	II

ORR, overall response rate; DCR, disease control rate; PFS, progression-free survival; OS, overall response; WT, wild-type; MT, mutated-type.

The efficacy of the combination of trastuzumab, a recombinant humanized monoclonal antibody that binds with high affinity and specificity to the extracellular domain of the HER2 receptor, and lapatinib, a reversible inhibitor of HER2/EGFR dual tyrosine kinase domains (8), was confirmed by the HERACLES study; eight (30%) of 27 patients with HER2 amplification achieved an objective response, and the progression-free survival (mPFS)was 21 weeks (95% CI 16 ~ 32) (2). However, acquired resistance occurred in almost all cases who ever experienced objective response in this study, same as the dilemma in the Mypathway study, a multiple basket study assessing the activity of trastuzumab and pertuzumab in mCRC.

It is known that EGFR and HER2 signal through the RAS/RAF/ERK pathway stimulating cell division, and the PI3K/PTEN/AKT pathway leading to cell growth and survival (8), and abnormalities in the downstream pathway can cause insusceptibility to targeted agents. However, the mechanisms of resistance to HER2-directed therapy are still unclear. In this case, we attempted to explore the mechanisms of resistance to trastuzumab and lapatinib by NGS. In breast cancer, activating mutations in PI3KCA and decreased expression of PTEN have been identified as potential mechanisms of resistance to trastuzumab and lapatinib (9). Moreover, Belli et al. demonstrated that aberrant alterations in MEK and PI3KCA can lead to anti-HER2 drugs’ resistance using models of HER2-amplified CRC (10). However, neither activating mutations of PI3KCA or MEK nor decreased expression of PTEN was detected in lung metastasis samples collected after failure of trastuzumab and lapatinib treatment in our case, although amplification of KRAS, loss of heterozygosity of INPP4B, and point mutation of *INPP4A* were observed. In the HERACLES trial, alterations in RAS/RAF were recorded in six (86%) of seven patients who were resistant to trastuzumab and lapatinib, suggesting a potential mechanism against to anti-HER2 therapy. Studies (11–13) showed that INPP4 inhibited tumor growth and infiltration by blocking the PI3K/AKT pathway, similar to the mechanism of action of the PTEN gene, thereby exerting tumor-suppressive effects, indicating alterations in INPP4 could be a new mechanism for resistance to trastuzumab and lapatinib. In this context, interestingly, the success of pyrotinib is elusive.

Pyrotinib (SHR-1258), a novel EGFR/HER2 dual TKI, can bind to the adenosine triphosphate binding site of HER2 and EGFR kinase regions irreversibly, thus preventing the formation of homogenous and heterodimers of HER2 and EGFR in tumor cells (4). In our case, albeit the resistance to trastuzumab and lapatinib, pyrotinib achieved an impressive clinical response with a longer PFS (8.5 months vs. 6 months). Smaller half-maximal inhibitory concentrations values against EGFR and HER2 of 5.6 and 8.1 nM than lapatinib’s those of 10.2 and 9.8 nM (14) and additional inhibition of HER-4 may be attributed to the superiority of pyrotinib in suppressing HER2-positive cells to lapatinib. In addition, trastuzumab, pertuzumab, and tucatinib only target HER2, while pyrotinib also has a strong inhibitory effect on EGFR. Moreover, pyrotinib shows high selectivity when tested against a panel of different kinases, including kinase insert domain receptor, c-Kit, platelet-derived growth factor receptor β, c-Src, and c-Met, in vivo (4). Accordingly, pyrotinib may exhibit various effects through several mechanisms different from those of previous anti-HER2 drugs, which can partly explain the remarkable response to pyrotinib of our case. Further studies are needed to elucidate these potential mechanisms.

Accumulated evidences support a less favorable response to anti-EGFR antibodies in patients with HER2 amplification (2, 15). However, we cannot tell if the benefit of survival comes from cetuximab or not in our single case. Sawada et al. reported that PFS was significantly shorter in mCRC patients with HER2-amplification than those with wild-type RAS/BRAF when treated with anti-EGFR therapy (mPFS 2.6 vs 6.0 months; hazard ratio [HR] 3.89; 95% CI 1.49~10.18) (16). In addition, Yonesaka et al.’s study showed that inhibition of HER2 can restore cetuximab sensitivity in vitro and in vivo (17). Together, these evidences suggest that HER2 amplification can be a potential mechanism of primary resistance to anti-EGFR antibodies. This is a thought-provoking phenomenon that may indicate the priority of anti-HER2 therapy to cetuximab or combined therapy of anti-HER2 drugs and cetuximab when treating HER2-amplified mCRC. Actually, some scholars have suggested that anti-HER2 therapy be moved towards an earlier line for mCRC patients with HER2 amplification (18), considering the superiority of anti-HER2 therapy and the inferiority of cetuximab in HER2 amplification setting. In addition, a concordance for HER2 positivity in the primary tumor, brain, and lung metastatic lesions was found in our case, suggesting that HER2 amplification occurred as an early molecular aberration that persists during tumor progression, and this provides a rationale for an earlier and continued application of anti-HER2 therapy. Although, the optimal timing and sequencing of anti-HER2 therapy remain to be elucidated.

Liquid biopsy data showing that RAS and EGFR mutant alleles decay after a minimum of 4 months from the last dose of anti-EGFR from Parseghian et al.’s study explains why anti-EGFR rechallenge may be active (19). Another evidence showed that inhibition of HER2 restored cetuximab sensitivity in vitro and in vivo (17), suggesting the combination of cetuximab and HER2 inhibitors represents a sensible therapeutic strategy for HER2-amplified patients. Considering that pyrotinib may reverse cetuximab’s resistance and that cetuximab may enhance the inhibition of EGFR by pyrotinib, the combination of pyrotinib and cetuximab was used after the failure of single pyrotinib in our patient as a rechallenge regimen. Finally, this combination has been shown to be effective and safe, with our patient obtaining a PFS of at least 4 months and being well tolerated. To some extent, our case demonstrates the feasibility of cetuximab in combination with pyrotinib.

A high TMB, as well as MSI-H as a predictive biomarker for response in mCRC, was revealed by previous studies, and patients carrying these characteristics are thought to be susceptible to immune checkpoint blockade (ICB) (20). The KEYNOTE-164 study evaluating pembrolizumab in MSI-H mCRC previously treated with more than one line of therapy showed that overall survival (OS) rates were 76% with a 41% PFS rate at 12 months (21). However, our patient developed PD after three cycles of pembrolizumab. A bioinformatics analysis of CRC data in The Cancer Genome Atlas found reduced infiltration of cytotoxic cells as well as reduced T helper 1-centric coordinated immune response cluster in KRAS-mutated CRC. This suggests that ICB may be less efficacious in this context (22). But it is unclear whether HER2 status can affect the efficacy of ICB. On this account, application of ICB in patients with resistance to HER2 blockade and KRAS mutations needs to be reconsidered.

It is worth noting that apatinib, a small molecule TKI targeting vascular endothelial growth factor receptor, achieved PR and a PFS of 12 months as a third-line treatment. A single-arm, phase II study of apatinib as a third-line treatment for mCRC reported that median mPFS and mOS were increased by 3.9 (95% confidence interval [CI] 2.1~5.9) and 7.9 months (95% CI 4.6~10.1+) respectively, suggesting that apatinib was efficacious as a third-line treatment for Chinese refractory mCRC (23). This needs more clinical trials to verify.

The limitation of our case lies in the lack of tissue or liquid biopsy results after the failure of pyrotinib, which makes it difficult to specify the gene status of tumor and explain the resistance to pyrotinib. If the patient’s disease progresses again, based on current genetic testing results and previous treatment regimens, mTOR inhibitors (e.g. everolimus) or FGFR inhibitors may be tried as salvage treatments.

## Conclusion

Efforts are ongoing to identify new clinically actionable oncogenic drivers other than MSI and RAS. HER2 is considered a promising target for mCRC. Herein, we described a patient with treatment-refractory HER2-amplified mCRC who experienced a prolonged response to pyrotinib after the resistance to trastuzumab and lapatinib. In conclusion, pyrotinib is active for mCRC patients with HER2 amplification in salvage treatment settings. Further studies are warranted to validate this result.

## Ethics Statement

Written informed consent was obtained from the individual for the publication of any potentially identifiable images or data included in this article.

## Author Contributions

J-YL: Guarantor of integrity of the entire study, manuscript editing. H-SL: Study concepts and design, manuscript preparation. L-LY: Collection and collation of medical records, manuscript editing. M-YZ: Literature research. KC: Patient follow-up. YC: Study concepts and design. All authors contributed to the article and approved the submitted version.

## Funding

This study was supported by the Sichuan Science and Technology Department Key Research and Development Project (2019YFS0539), 1.3.5 Project for Disciplines of Excellence, West China Hospital, Sichuan University (ZYJC18022) and the National Clinical Research Center for Geriatrics (West China Hospital, Z2018B12).

## Conflict of Interest

The authors declare that the research was conducted in the absence of any commercial or financial relationships that could be construed as a potential conflict of interest.
