# Supplementary material for: Remarkable Response of EGFR- and HER2-Amplified Metastatic Colon Cancer to Pyrotinib After Failed Multiline Treatments: A Case Report and Literature Review
Source: Front Oncol. 2020 Oct 26;10:548867. doi: 10.3389/fonc.2020.548867 (PMC7649345; doi:10.3389/fonc.2020.548867)
Supplement: Supplementary file 1 [file Table_1.docx]

**Supplementary Table 1** Main Ongoing clinical trials evaluating anti-HER2 treatments in mCRC

| NCT Number/EudraCT Number | Title | Interventions | Phase | Number | Dates | Locations |
| --- | --- | --- | --- | --- | --- | --- |
| NCT03418558 | Study of Trastuzumab- emtansine in Patients With HER2-positive Metastatic Colorectal Cancer Progressing After Trastuzumab and Lapatinib | • Drug: Trastuzumab emtansine | II | 13 | 2015/7/8 | Italy |
| NCT03457896 | Study of Neratinib + Trastuzumab or Neratinib + Cetuximab in Patients With KRAS/NRAS/BRAF/PIK3CA Wild-Type Metastatic Colorectal Cancer by HER2 Status | • Drug: Trastuzumab  • Drug: Cetuximab  • Drug: Neratinib | II | 35 | 2018/5/18 | United States |
| NCT03843749 | Pyrotinib in Combination with Trastuzumab in Treatment- refractory, HER2-positive Metastatic Colorectal Cancar | • Drug: Pyrotinib | NA | 30 | 2019/7/1 | China |
| NCT03043313 | Tucatinib Plus Trastuzumab in Patients with HER2+ Colorectal Cancer | • Drug: Trastuzumab  • Drug: Tucatinib | II | 110 | 2017/6/23 | United States |
| NCT03365882 | S1613, Trastuzumab and Pertuzumab or Cetuximab and Irinotecan Hydrochloride in Treating Patients with Locally Advanced or Metastatic HER2/Neu Amplified Colorectal Cancer That Cannot Be Removed by Surgery | • Drug: Cetuximab  • Drug: Irinotecan Hydrochloride  • Drug: Pertuzumab  • Drug: Trastuzumab | II | 130 | 2017/10/9 | United States |
| 2012-002128-33 | Open-Label, Phase II Study of Trastuzumab in Combination with Lapatinib or Pertuzumab in Combination with Trastuzumab in Patients with HER2-positive Metastatic Colorectal Cancer: the HERACLES Trial (HER2 Amplification for Colo-rectal Cancer Enhanced Stratification) | • Drug: Trastuzumab  • Drug: Pertuzumab  • Drug: Lapatinib | II | 27 | 2012/7/23 | Italy |

NA, not applicable.

Accessed at *clinicaltrials.gov* and *clinicaltrialsregister.eu* on 2020/8/20.
